# Supplementary material for: Pivoting in the pandemic: a qualitative study of child and adolescent psychiatrists in the times of COVID-19
Source: Child Adolesc Psychiatry Ment Health. 2021 Jun 21;15:32. doi: 10.1186/s13034-021-00382-6 (PMC8216090; doi:10.1186/s13034-021-00382-6)
Supplement: Supplementary file 1 — Additional file 1: Appendix S1. Sensitizing questions. [file 13034_2021_382_MOESM1_ESM.docx]

**Appendix S1.
Sensitizing questions**

Below are a series of questions that will form the ground for our one-hour interview in two weeks. Please feel free to reflect on any that stand out to you. You do not have to answer every question, and you do not need to prepare formal answers -- these are just to encourage reflection prior to the interview.

1. In what ways, if any, is COVID-19 impacting your life?
2. When did “the time of COVID-19” start for you, and why then?
3. How has the structure of your day been altered, if at all, due to COVID-19 and social distancing?
4. In what ways, if any, has your daily response to the pandemic changed over time?
5. What shifts, if any, have you noticed in yourself (behavioral, emotional, cognitive, interpersonal, etc.) and in your relationships (patients, families, students, colleagues, etc.) during this time?
6. What effects of COVID-19 on your work/home have been easier and more difficult to manage?
7. How do you envision the field of psychiatry “emerging” from this time period, after COVID-19? What kind of culture would you want to create for child psychiatry moving forward?
